# Fatigue and Syncope Caused by Right Ventricular Perforation by a Pacemaker Lead

**DOI:** 10.7759/cureus.22634

**Published:** 2022-02-26

**Authors:** Jehanzeb A Khan, Shuja Abdul Karim Khan

**Affiliations:** 1 Internal Medicine, University of Oklahoma Health Sciences Center, Oklahoma City, USA; 2 Medicine, Dow University of Health Sciences, Karachi, PAK

**Keywords:** pacemaker lead displacement, permanent pacemaker, pacemaker extraction, pacemaker lead perforation, economic burden of healthcare, right ventricular perforation, permanent pacemaker (ppm) complication

## Abstract

Cardiac devices with intra-ventricular leads are associated with a risk of perforation. This can be a diagnostic challenge given that there is a whole spectrum of clinical presentation from incidental discovery on imaging to large effusions and tamponade. Here we present a case where a patient with permanent pacemaker for complete heart block presented with worsening fatigue that deteriorated to syncopal episodes. Electrocardiogram revealed bradycardia with junctional escape and imaging revealed the tip of the right ventricular lead beyond the ventricular wall. The lead was replaced under fluoroscopic guidance without the need for surgical intervention and the patient was ready for discharge on post-procedure day one. Replacement under fluoroscopic guidance is not only safe, but also enables early discharge, which reduces the burden on health care facilities as well as minimizes the patient's number of days lost in the hospital.

## Introduction

With the continued growth of medical sciences, more and more patients requiring advanced therapies, including pacemakers, are identified and treated appropriately. Generally, these devices are very safe but they can be associated with complications, perhaps the most concerning of which is lead perforation through the ventricular wall. The risk of right ventricular (RV) perforation is reported at 0.1-0.8% [1]. While some cases of lead perforations can be asymptomatic and incidentally found on imaging, they can present with chest pain, loss of capture with bradycardia associated symptoms, and in severe cases, pericardial effusion and tamponade [2-5]. Most of the lead perforations occur early after implantation [6,7]. However, late perforation is also possible with some cases reported as late as 4.8 years after the procedure, hence highlighting that timing of device placement should not preclude the diagnosis of lead perforation [8]. The management of perforations is usually surgical lead removal, but removal by traction under fluoroscopic guidance has also shown to be safe and effective [9,10]. As these devices become more readily available to the general population, it is important for the providers to be aware of possible complications. It is also imperative to identify how these complications can be addressed in the most efficient manner, to ensure that the device-related complications do not become a large burden on health care resources. Here we present a case of a 69-year-old female with RV lead perforation who presented with worsening fatigue and three syncopal episodes. She underwent lead extraction via traction under fluoroscopy and placement of a new RV lead. Fluoroscopy-guided minimally invasive procedure enabled the patient to be discharged the day after her procedure.

## Case presentation

**Figure 1:**
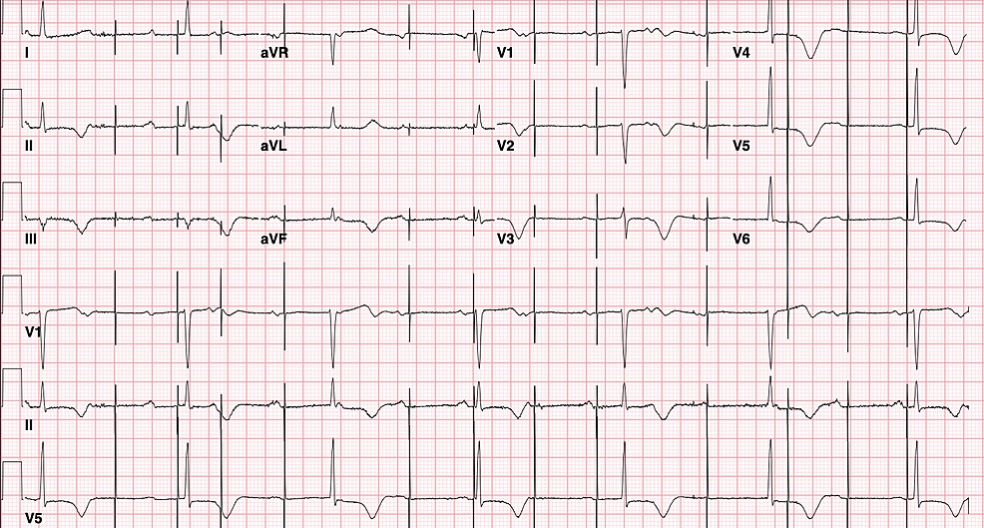
EKG showing failure to sense and failure to capture with junctional escape rhythm

A 69-year-old female with a permanent pacemaker for complete heart block and coronary artery disease with stents to the first diagonal artery (D1) was admitted with concerns of worsening fatigue and three syncopal episodes. About a year prior to her current presentation, she underwent a workup for fatigue and malaise. Cardiac evaluation at that time showed a complete heart block as well as in-stent restenosis of her previous D1 vessel. She subsequently underwent a dual-chamber permanent pacemaker placement and percutaneous coronary intervention with drug-eluting stent to the M1 stent. Post-procedurally, she did well for the first four months but then started to experience worsening fatigue and malaise. She also had three syncopal episodes, which were described as a sudden loss of consciousness without any preceding alarming signs or symptoms. She subsequently presented to the ER and at the time of admission, she was found to have heart rates between 30-60 beats per minute. Her blood pressure was 130-150/40-50mmHg. Her labs showed a hemoglobin of 10 g/dL, which was stable compared to the previous year. Her thyroid-stimulating hormone (TSH) was 2.1 IU. Electrocardiogram (EKG) showed intermittent failure to sense and failure to capture, with junctional escape rhythm (Figure 1).

**Figure 2:**
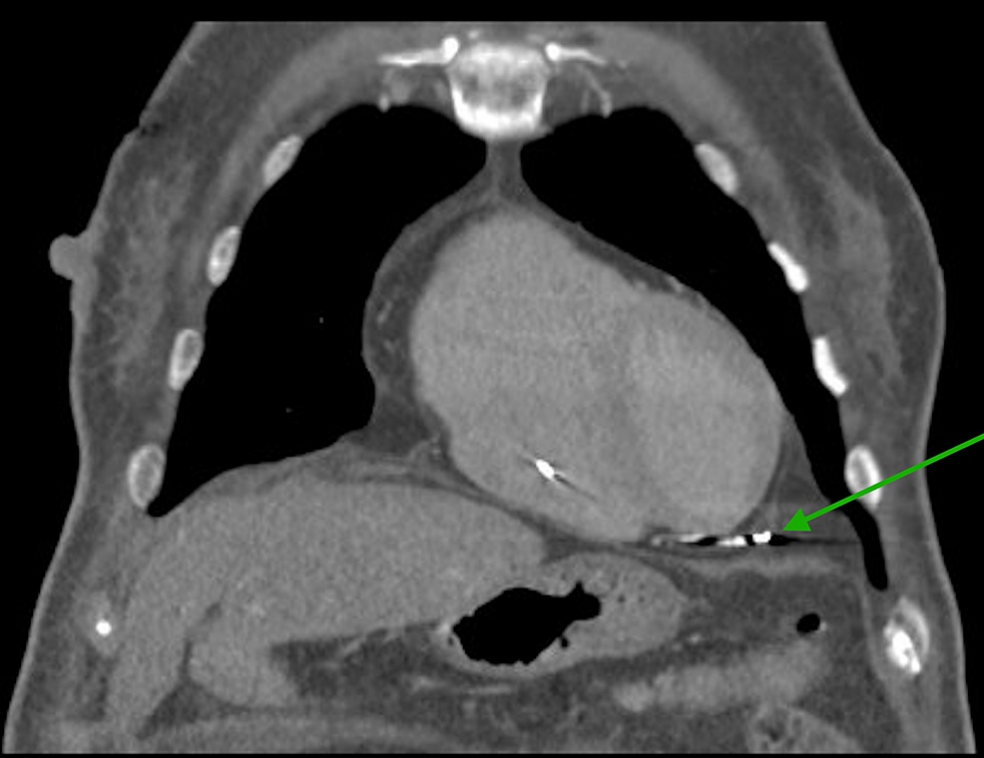
CT scan of the chest, coronal slice, showing the RV lead beyond the ventricular wall RV: right ventricular

Device interrogation revealed a pacing threshold of 6.5V at 2.0ms compared to 1.5V at 2.0ms from her last interrogation seven months ago. A chest X-ray from the ER showed concerns of the RV lead beyond the ventricular wall. A subsequent CT scan of the chest showed the RV lead perforating the RV with its tip projecting into the pericardial space (Figure 2).

**Figure 3:**
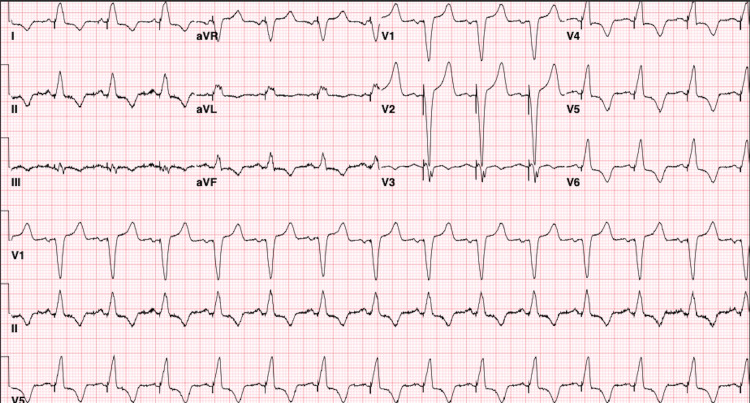
Post-procedural EKG showing atrial sensing and ventricular pacing

A transthoracic echocardiogram (TTE) showed a trivial pericardial effusion. After discussion with the patient, she decided to proceed with lead replacement. Under general anesthesia, her left pectoral pocket was opened and, using fluoroscopy, a new lead was introduced into the RV and placed along the antero-septal part of the right ventricle. After the new lead was noted to be capturing, the old lead was removed by simple traction, and the procedure was uncomplicated. Next morning, the device was interrogated, and the patient was seen to be pacing at the desired rate without concerns of loss of capture. EKG showed an atrial sensed and ventricular paced rhythm (Figure 3). The patient also reported an improvement in her sense of fatigue and tiredness. Her only concern was minimal tenderness over the pectoral pocket. Chest X-ray and TTE were both non-concerning, and the patient was ready for discharge.

## Discussion

As cardiovascular sciences continue to grow with evidence-based medicine, more and more people with symptomatic bradyarrhythmia and life-threatening heart blocks get access to pacemaker devices. While generally these devices are safe, they are associated with complications like infections, device malfunction or, in rare cases, ventricular wall perforation. The rates of RV lead perforation in permanent pacemakers (PPM) and implantable cardioverter defibrillator (ICD) are reported at 0.1-0.8% and 0.6-5.6%, respectively [1]. Similarly, in the OPTIMUS registry (that evaluated the long-term performance of SJM leads), RV perforation was observed in 0.33% of the cases with ICD leads and 0.5% of the cases with PPM leads [11].

RV lead perforations can have a spectrum of clinical presentations. While cases have been described where the perforating lead was found incidentally on imaging [2], they often present with chest pain and shortness of breath [3]. Occasionally, they can present with failure to capture and subsequently problems associated with bradyarrhythmia or heart blocks [4]. Sometimes the perforated lead can cause large pericardial effusions which can present with tamponade [5]. As highlighted in the case above, presenting complaints can be very vague and, non-specific symptoms like fatigue might be the only reported symptoms that can later deteriorate into syncopal episodes. This highlights the need for all providers to be very cautious when caring for patients with intraventricular devices and leads, and even in the absence of symptoms or nonspecific symptoms, the index of suspicion of lead malfunction and possible perforation should be high.

Most of the lead perforations occur early after implantation [6], as per some studies about 2/3rd of the cases of RV lead perforation presented in the first three months [7]. This case highlights another important point that delayed lead perforation is also possible, and irrespective of the time from device placement, the providers when caring for patients with intra-cardiac devices should always bear in mind the possibility of a perforating lead.

While previously perforating leads were addressed only surgically with double-patch sutures, studies have shown that in cases without large pericardial effusion, transvenous removal of the perforated lead under fluoroscopic guidance is not only effective, but also very safe with a low risk of complications [7,9,10]. This case highlights the above and reflects how with close overnight monitoring for complications like pneumothorax, hemothorax, pericardial effusion and tamponade, the patients can be safely discharged home after overnight observation. As pacemaker devices become more prevalent, it is essential that we identify more effective ways of addressing device-related complications. This can eventually result in decreased cost and decreased burden on healthcare facilities and improve patients' experience and reduce the number of days lost in the hospital.

## Conclusions

While devices with intra-ventricular leads are generally safe, they are associated with their risk of complications including infections, lead malfunction or ventricular wall perforation. This case highlights how lead perforations can have a variety of clinical presentations, from being totally asymptomatic to presenting with failure to capture and pace the ventricles with symptoms associated with bradyarrhythmia or heart blocks, and in the most severe cases, large pericardial effusion, and tamponade. Therefore, physicians treating patients with cardiac devices should generally have a very low threshold to evaluate for pacemaker dysfunction even if the patient is presenting with non-specific symptoms, and this needs to be independent of the time from device placement, as most leads perforate the RV during the first couple of months, but delayed presentation is also possible.

Another point that this case brings attention to is that lead perforations that once required surgical intervention can now be safely treated via a transvenous approach. While this is minimally invasive, it is also very effective and safe and can enable earlier discharge from the hospital that will not only alleviate the burden from health care facilities but also help with patient satisfaction.
